# Body dissatisfaction, drug use, and associated factors among adolescents in three Brazilian cities*

**DOI:** 10.1590/1518-8345.6163.3663

**Published:** 2022-09-21

**Authors:** Ryvanne Paulino Rocha, Patrícia Paiva de Oliveira Galvão, Zila van der Meer Sanchez, Lidiane Nogueira Rebouças, André Ribeiro de Castro, Luís Eduardo Soares dos Santos, Mariana Cavalcante Martins, Patrícia Neyva Da Costa Pinheiro, Neiva Francenely Cunha Vieira, Fabiane do Amaral Gubert

**Affiliations:** 1 Universidade Federal do Ceará, Departamento de Enfermagem, Fortaleza, CE, Brasil.; 2 Bolsista da Coordenação de Aperfeiçoamento de Pessoal de Nível Superior (CAPES), Brasil.; 3 Universidade Federal de São Paulo, Departamento de Medicina Preventiva da Escola Paulista de Medicina, São Paulo, SP, Brasil.; 4 Bolsista do Conselho Nacional de Desenvolvimento Científico e Tecnológico (CNPq), Brasil.; 5 Governo do Estado do Ceará, Secretaria Executiva Sobre Drogas do Ceará, Fortaleza, CE, Brasil.

**Keywords:** Body Dissatisfaction, Adolescent, Alcohol Drinking, Drug Abuse, Health Promotion, Students

## Abstract

**Objective:**

analyze the association between drug use and body dissatisfaction among adolescents in three Brazilian cities.

**Method:**

cross-sectional study, using a nested randomized controlled trial to evaluate the drug use prevention program *#TamoJunto2.0* of the Ministry of Health in Brazilian schools. The sample consisted of 5,213 students from 73 schools in three Brazilian cities. The outcome body satisfaction was analyzed using the Stunkard scale and the explanatory variables were drug use and sociodemographic data.

**Results:**

the adolescents were between 12 and 14 years old; about 69.9% of them reported body dissatisfaction, and 35.67% used alcohol in the previous year. Dissatisfaction due to overweight was higher among girls (41.5%) and dissatisfaction due to underweight was higher among boys (33.1%). Adolescents who used marijuana were 39% (OR=1.39) more likely to feel dissatisfied due to underweight and being a girl increased the chances of feeling dissatisfied due to overweight by 24% (OR=1.24).

**Conclusion:**

the levels of body dissatisfaction deserve attention in hebiatric nursing care and reinforce the importance of educational strategies addressing body image and drug use, relating them to the various subjective attributes that can affect the health of adolescents, whether in the community or at school.

## Highlights

(1) High prevalence of body dissatisfaction related to overweight.

(2) Girls presented 24% more chances of feeling body dissatisfaction due to overweight.

(3) Marijuana use in adolescence can increase by 39% body dissatisfaction due to underweight.

(4) Last year, about 35.7% of adolescents had already consumed alcohol.

(5) Health promotion and stimulation of positive perceptions of self-image.

## Introduction

Body dissatisfaction in adolescence refers to a divergence in the perception of the real body and the ideal body, characterized by a negative view of oneself^1^, which can have implications to physical, mental and social well-being in this accelerated phase of development^2^.

Some factors, such as physical inactivity, unprotected sex, overweight, eating disorders, alcohol consumption, smoking, and drug use may be related to body dissatisfaction in adolescence, which may lead to irreparable health outcomes in adulthood^2^ ^-^ ^3^. Adolescents who face changes in the perception of their body image are prone to the consumption of psychoactive substances as strategies to relief and protection against stress, anxiety and anguish^4^.

The use of licit and illicit drugs is a growing concern in several countries, with alcohol and marijuana being the most consumed drugs in the world^5^. In Brazil, the National School Health Survey (PeNSE) (2019), which is the largest school survey ever conducted, shows that 63.3% of students said they had already consumed alcohol, and more than 22.6% had tried smoking. Among the illicit drugs, the use of marijuana prevailed^6^; and in contrast to national indices that indicate a reduction in smoking in Brazil, an increasing use of electronic cigarettes and hookahs was observed^7^. In addition, the adoption of unhealthy behaviors and without proof of safe efficacy for weight control^8^ ^-^ ^9^ may be associated with the use of cigarettes and marijuana and body dissatisfaction^10^.

The literature shows evidence of a strong association between body dissatisfaction and use of psychoactive substances, especially among female individuals^11^ ^-^ ^12^. This relationship of non-acceptance of the body may also be related to the different sociocultural requirements in body standardization, making it difficult to accept the diversity that differs from this established model^13^. The relationships between body image, culture, body identity, media standard, and the contextualization of these factors in time and space are complex, affecting individual behavior in search of the beauty standard^14^.

In view of the above, the level of body satisfaction and its related factors deserve attention in adolescence as a public health and mental health problem^2^ ^-^ ^3^, and the literature has few studies that analyze the association between body image and abuse of substances, such as marijuana, inhalants, weight-loss drugs, and the practice of binge drinking (BD), mainly performed by nurses. Studies on body satisfaction are usually produced in high-income countries; then, an important gap is observed in knowledge about adolescents in Latin America, a region with many social and economic differences and cultural factors that influence body appreciation^15^, in addition to the context of different socioeconomic classes. In Brazil, a continental country, there are different ways of “being a teenager,” mainly in the study population of teenagers from different regions.

With a better understanding of the factors related to body dissatisfaction among adolescents, it is possible to identify situations of vulnerability and guide specific public actions and policies to prevent risk behaviors related to body image, whether in drug use or other negative health outcomes. In this context, the possibility of nurse’s actions is highlighted, as well as the strengthening of government programs that allow a focus on adolescent health in primary care and other levels of health care. Therefore, this study aimed to analyze the association between drug use (smoking, over-the-counter weight-loss drugs, marijuana, inhalants, cocaine, alcohol, and excessive alcohol consumption) and body dissatisfaction among adolescents in three Brazilian cities.

## Method

### Study design

This study used a sample of a randomized controlled trial (RCT) whose initial objective was to evaluate the effectiveness of a drug use prevention program for 8th grade adolescents^16^. Then, the RCT with two parallel groups (intervention and control) was conducted with among 8th grade students from public schools in three Brazilian cities (São Paulo/SP, Fortaleza/CE, and Eusébio/CE). This study was registered in the Brazilian Registry of Clinical Trials - REBEC, of the Ministry of Health (number RBR-8cnkwq) and approved by the research ethics committee of the Federal University of São Paulo (protocol 2.806.30).

### Context

The selection of cities and the articulation with the health care secretariats, education at the municipal and state levels for the implementation of the *#TamoJunto2.0* program, and the study were conducted by the Coordination of Mental Health, Alcohol and Other Drugs of the Ministry of Health, responsible for the program implementation.

Then, this multicenter study was performed in 73 public schools. The cities participating in this study were São Paulo (SP), the most populous city in the country with an estimated population of over 12 million and with a Human Development Index (HDI) of 0.805; Fortaleza (CE), capital of the State of Ceará, with around 2.7 million inhabitants and an HDI of 0.754; and Eusébio (CE), located in the metropolitan region of the capital of Ceará, with 55,000 inhabitants and an HDI of 0.701.

The only school selection criterion was to have 8th grade students, as this grade is indicated for the application of the *#Tamojunto2.0* program, and the selection was made according to the size of the cities) using the national list of registrations of the National Institute of Educational Studies and Research Anísio Teixeira (INEP). All 8th grade students from each school were included in the study.

### Participants

The sample consisted of 5,213 students from 205 classes of 8th graders^16^. Participants with some type of reduced cognitive or physical capacity to fill out the instrument had a trained researcher to help them in a private place at the school. The adolescents were identified by a confidential code and, after filling the instrument, they placed it in an unidentified envelope that was sealed in front of them.

The school acceptance rate was 93.6% (78 schools were invited) and, considering the enrollment records, 6,993 students were expected in the participating classes; however only 23% absences were reported, related to classes that no longer existed at the time of the study or were grouped together due to school renovation, or the adolescents were no longer attending the institution. Only 123 students refused to participate, generating a refusal rate of 2.2%. More information about the study design and sampling procedures are described in previously published articles^16^ ^-^ ^17^.

### Data collection/instrument

Data were collected from February to March 2019 using an anonymous self-report questionnaire, applied by trained researchers in the classroom, without the presence of the teacher or another school employee. The questionnaire was adapted from the instrument previously developed and tested by the European Drug Abuse Prevention Trial (EU-Dap) and used in previous studies assessing the effectiveness of *Unplugged* ^18^. A version translated and adapted to Brazilian Portuguese^19^ was used, with some questions replaced by items from two questionnaires widely used in several Brazilian studies that assess students: a questionnaire from the World Health Organization (WHO), used in the VI Brazilian Survey on Drug Use among Students^20^, and the questionnaire of the National Student Health Survey, used by the Brazilian Ministry of Health^21^.

The final questionnaire used in this study has been validated in Brazil^22^. It has modules addressing sociodemographic data and presents information about when (month, year, and age) the adolescent used the following drugs: alcohol, smoking, marijuana, inhalants, cocaine, amphetamines, benzodiazepines, and crack cocaine; practice of binge drinking (consumption of 5 or more alcoholic drinks in a short period), as well as questions assessing body satisfaction.

### Study variables

The outcome variable “body satisfaction” was analyzed through two questions about body perception and satisfaction based on the Stunkard scale, an instrument that has been widely used with adolescents^23^ ^-^ ^24^ and which contains nine male body and nine female body silhouette figures. The participants were asked two direct questions about their body image:

- How do you see yourself? - variable called “real self-image”. The participant should mark the silhouette that best represented his/her own image at that moment;
- How would you like to look? - variable called “ideal self-image”. The participant should mark the ideal image he/she would like to have.

Then, by subtracting the value of the figure representing the real self-image from the value of the figure representing the ideal self-image, the resulting values ranged from -8 to +8. According to the result of the operation, every participant was classified as: satisfied (result equal to zero), dissatisfied due to underweight (plus value), or dissatisfied due to overweight (minus value)^23^.

The explanatory variables analyzed in the study were: 1) Drug use by adolescents (yes or no) in the last year: smoking, over-the-counter weight-loss drugs, marijuana, inhalants, cocaine, alcohol and excessive alcohol consumption (consumption of 5 or more alcoholic drinks over a two-hour period); 2) Sociodemographic data: sex, age, city, and socioeconomic status (the latter was evaluated using the scale of the Brazilian Association of Market Research Companies (ABEP)^25^, which takes into account the education of the head of the family and the goods and services consumed, with scores ranging from 1 to 100 or categories from A to E; higher scores indicate better economic status, and socioeconomic classes are ranked A (highest) to E (lowest). According to ABEP, classes D/E may appear grouped. All variables used in this study were collected in the beginning of the study (baseline) and, therefore, were not randomized or influenced by the intervention.

### Data treatment and analysis

Analyses were performed using weighted data to correct for uneven probabilities of sample selection. The sample weights considered school as the main sampling unit, with stratification by city, total number of students expected in each class, students present on the day of the analysis, and total expected universe in each municipality according to the national registry (INEP). For descriptive statistics of weighted percentages (% by weight), Stata 16 was used, with svy commands.

To assess the agreement between real self-image and ideal self-image, this study determined correspondences between silhouette categories and each nutritional status category: underweight (silhouettes 1, 2, 3), eutrophy (silhouettes 4, 5, 6), and overweight (silhouettes 7, 8, 9). The agreement between real self-image and ideal self-image was assessed using Kappa statistics in Stata 16.

As a large amount of data related to body dissatisfaction was not provided by the adolescents when answering the form, missing data were imputed in Mplus version 8.0 by multiple imputation using a sequential imputation approach, that is, multiple imputations were performed using the Bayes estimation and an unconstrained variance-covariance model to assign the missing values^26^. The following variables were used in the unconstrained model: group, school, city, sex, age, drug use (alcohol, excessive consumption of alcohol, smoking, inhalants, marijuana, cocaine, and weight-loss drugs), and ABEP classification. Fifty sets of imputed data were generated.

Then, univariate and multivariate multinomial logistic regressions were performed in Mplus 8.0, with the explanatory variables (drug use, city, ABEP score, sex, and age) affecting the outcome measurement (body satisfaction). An initial multivariate regression model was considered, including the explanatory variables with a value of p≤0.20 in the univariate regression, and then a retroactive procedure was manually performed to remove the explanatory variables with p>0.05, eliminating the variable with the highest p value, one by one, in order to check for correlation between the explanatory variables^27^ and thus obtain a final model for each response variable (dissatisfaction due to underweight and dissatisfaction due to overweight), the respective odds ratio (OR), 95% confidence interval (CI), and p values. The significance level was 5%.

### Ethical aspects

This study observed all ethical precepts of Resolution n. 466/2012, of the National Health Council (CNS), and was approved by the Research Ethics Committee of the Federal University of São Paulo, under approval n. 2.806.301 and CAAE: 91614918.9.0000.5505 of 2018. All adolescent sand school principals signed an informed consent form (ICF). The parents/guardians of adolescents were not required to sign an ICF because this is a program created by the Ministry of Health that has operated since 2013 as a routine action in several public schools in some Brazilian cities via Programa Saúde na Escola - PSE (School Health Program) by the teacher during class time and within the academic year. All participants were instructed about the study objectives and the possibility of withdrawing at any stage of the study.

## Results

Table 1 shows the sociodemographic data of participants, with gender balance between boys (50.06%) and girls (49.94%), predominance of adolescents aged 12 to 14 years (89.27%), and mean age of 13 .23 years (SD=0.85). The most common socioeconomic stratum based on ABEP was category C (54.03%).

When asked about the ideal self-image, most adolescents (51.65%) chose silhouette figures 1 to 3 that indicate underweight. Almost 70% of adolescents reported body dissatisfaction, mostly due to overweight (38.44%). Alcohol was the most frequent drug among the adolescents in the last year (35.67%), as indicated in Table 1.

**Table 1:** Distribution of participants according to sociodemographic variables, body image, and drug use (N=5213). Brazil, 2019

VARIABLES		N	%	95%CI^*^
**City**				
	São Paulo	2376	58.53	[54.78; 62.19]
	Fortaleza	2051	30.37	[27.53; 33.36]
	Eusébio	786	11.10	[10.08; 12.22]
**Sex**				
	Male	2578	50.06	[49.04; 51.08]
	Female	2573	49.94	[48.92; 50.96]
**Age (years)**				
	Mean ± SD^†^	13.23±0.85		
	12-14	4648	89.27	[88.22; 90.23]
	15-17	565	10.73	[9.77; 11.78]
**SES** ^‡^				
	Mean ± SD^†^	24.75±9.19		
	A (45-100)	179	3.91	[3.41; 4.48]
	B (29-44)	1282	27.19	[25.08; 29.40]
	C (17-28)	2809	54.03	[52.50; 55.55]
	D/E (1-16)	884	14.87	[13.45; 16.42]
**Real self-image (How do you see yourself?)** ^§^				
	Silhouettes 1 to 3	2093	50.03	[48.88; 51.18]
	Silhouettes 4 to 6	2029	48.42	[47.27; 49.57]
	Silhouettes 7 to 9	71	1.55	[1.30; 1.86]
**Ideal self-image (How would you like to look?)** ^§^				
	Silhouettes 1 to 3	2086	51.65	[50.61; 52.69]
	Silhouettes 4 to 6	1940	48.10	[47.05; 49.15]
	Silhouettes 7 to 9	10	0.25	[0.16; 0.38]
**Body satisfaction** ^§^				
	Satisfied	1146	30.11	[29.11; 31.13]
	Dissatisfied due to underweight	1226	31.45	[30.25; 32.67]
	Dissatisfied due to overweight	1493	38.44	[37.51; 39.38]
**Drug use in previous year**				
	Alcohol	1758	35.67	[34.63; 36.73]
	Binge drinking	987	19.28	[18.45; 20.15]
	Smoking	320	5.98	[5.43; 6.59]
	Inhalants	478	9.34	[8.69; 10.02]
	Marihuana	281	5.67	[5.12; 6.27]
	Cocaine	19	0.35	[0.26; 0.47]
	Weight-loss drugs	48	1.03	[0.82; 1.28]

^*^CI = Confidence interval; ^†^SD = Standard deviation; ^‡^SES = Socioeconomic classification according to the Brazilian Association of Market Research Companies (ABEP); ^§^Stunkard scale

Table 2 shows data about body satisfaction; the responses provided by the boys showed a balance, while most girls were dissatisfied due to overweight (41,5%).

**Table 2:** Distribution of adolescents according to body satisfaction and variables of interest (sex, age, socioeconomic status, city, and drug use) (N=5213). Brazil, 2019

VARIABLE		Body satisfaction						
		Satisfied		Dissatisfied due to underweight		Dissatisfied due to overweight		P*
		%	95%CI^†^	%	95%CI^†^	%	95%CI^†^	
**SEX**								
	Male	31.2	[29.8;32.6]	33.1	[31.4;0.34.9]	35.7	[34.3;37.1]	<**0.001**
	Female	28.6	[27.4;29.9]	29.9	[28.4;31.5]	41.5	[40.0;43.0]	
**AGE**								
	12-14	29.9	[28.8; 31.0]	31.1	[29.9; 32.4]	39.0	[38.0; 39.9]	**0.097**
	15-17	31.9	[28.1; 36.0]	33.7	[29.9; 37.3]	34.4	[30.6; 38.4]	
**ABEP** ^‡^								
	A	34.1	[28.6; 40.1]	21.7	[15.1; 30.2]	44.2	[38.8; 49.7]	<**0.001**
	B	29.0	[27.1; 31.3]	28.4	[26.4; 30.4]	42.6	[40.2; 45.0]	
	C	30.8	[29.5; 32.1]	32.5	[31.2; 33.7]	36.8	[35.5; 38.0]	
	D/E	29.3	[27.0; 32.0]	35.1	[32.5; 37.7]	35.6	[33.5; 37.8]	
**CITY**								
	Eusébio	26.9	[25.3; 28.6]	31.5	[30.0; 33.2]	41.6	[39.7; 43.5]	<**0.001**
	Fortaleza	29.6	[28.1; 31.1]	33.4	[31.9; 35.0]	37.0	[35.6; 38.4]	
	São Paulo	31.5	[29.5; 32.7]	30.4	[28.5; 32.3]	38.5	[37.1; 40.0]	
**DRUG USE**								
**Alcohol**								
	No	31.2	[29.9; 32.5]	30.9	[29.5; 32.3]	37.9	[36.8; 39.1]	**0.031**
	Yes	28.2	[26.4; 30.1]	32.1	[30.5; 33.9]	39.7	[37.9; 41.4]	
**Binge drinking**								
	No	30.8	[29.6; 32.0]	30.9	[29.5; 32.4]	38.3	[37.2; 39.4]	**0.036**
	Yes	27.1	[24.7; 29.7]	33.6	[31.2; 36.0]	39.3	[36.9; 41.7]	
**Smoking**								
	No	30.3	[29.3; 31.3]	31.0	[29.7; 32.3]	38.7	[37.7; 39.7]	**0.053**
	Yes	26.9	[22.6; 31.6]	36.3	[32.8; 40.0]	36.8	[32.7; 41.2]	
**Inhalants**								
	No	30.8	[29.8; 31.9]	30.8	[29.5; 32.1]	38.4	[37.4; 39.3]	<**0.001**
	Yes	23.1	[20.8; 25.5]	35.5	[32.7; 38.4]	41.4	[38.1; 44.7]	
**Marijuana**								
	No	30.2	[29.2; 31.3]	31.0	[29.7; 32.3]	38.8	[37.8; 39.8]	**0.007**
	Yes	26.1	[22.2; 30.4]	37.7	[33.9; 41.5]	36.2	[32.4; 40.2]	
**Cocaine**								
	No	29.9	[28.9; 31.0]	31.6	[30.3; 32.8]	38.5	[37.5; 39.4]	**0.725**
	Yes	33.8	---	25.1	---	41.1	---	
**Weight-loss drugs**								
	No	30.2	[29.2; 31.2]	31.4	[30.2; 32.6]	38.4	[37.3; 39.4]	**0.013**
	Yes	14.4	[8.6; 23.0]	38.2	[26.5; 51.5]	47.4	[35.7; 59.4]	

^*^Test ꭓ^2^; ^†^CI = Confidence interval; ^‡^ABEP = Brazilian Association of Market Research Companies; ^§^Absent standard error due to stratum with single sampling unit

A prevalence of dissatisfaction due to overweight was also observed in the three cities, at all socioeconomic levels, and among all adolescents who reported consumption of alcohol or another drug, except among those who reported marijuana use, who had a higher percentage of dissatisfaction due to underweight.

Regarding the agreement between real and ideal self-image, a high level of agreement was observed among boys in the eutrophic category (68.32%), with a statistically significant kappa value (0.234) (p<0.001), and satisfactory agreement between the real self-image (how the boys saw themselves) and the ideal self-image (how they would like to look). Among girls, a high level of agreement was observed in the underweight category (63.44%), with a statistically significant kappa value (0.072) (p<0.001), but indicating weak agreement (Table 3).

**Table 3:** Agreement between real self-image and ideal self-image of adolescents according to sex (N=5213). Brazil, 2019

VARIABLE	Ideal self-image*									
	Boys									
	Underweight		Eutrophy		Overweight		Total			
Real self-image^†^	n	%	n	%	n	%	n	%	Kappa	p^‡^
Underweight	500	**55,43**	397	44,01	5	0,55	902	100	0,234	<0,001
Eutrophy	293	31,57	634	**68,32**	1	0,11	928	100		
Overweight	14	32,56	26	60,47	3	**6,98**	43	100		
Total	807	43,09	1057	56,43	9	0,48	1873	100		
**VARIABLE**	**Ideal self-image** ^*^									
	**Girls**									
	**Underweight**		**Eutrophy**		**Overweight**		**Total**			
**Real self-image** ^†^	n	%	n	%	n	%	n	%	**Kappa**	**p** ^‡^
Underweight	635	**63,44**	366	36,56	0	0,00	1001	100	0,072	<0,001
Eutrophy	521	56,08	407	**43,81**	1	0,11	929	100		
Overweight	15	60,00	10	40,00	0	**0,00**	25	100		
Total	1171	59.90	783	40,05	1	0,05	1955	100		

*How would you like to look?; ^†^How do you see yourself?; ^‡^p value

Underweight: silhouette figures 1-3; eutrophy: silhouette figures 4-6; overweight: silhouette figures 7-9.

According to Table 4, being a girl increases by 24% (OR=1.24) the chances of body dissatisfaction due to overweight when compared to boys, and every 1 point according to ABEP increases by 1% (OR=1.01) the chances of body dissatisfaction due to overweight. In contrast, adolescents who use marijuana are 39% (OR=1.39) more likely to feel body dissatisfaction due underweight than those who did not use marijuana.

**Table 4:** Multinomial logistic regression to identify factors associated with body dissatisfaction due to underweight and overweight in a sample of Brazilian students (N=5213). Brazil, 2019

		Univariate regression						Multivariate regression					
		Dissatisfaction due to underweight ****versus* satisfied***			Dissatisfaction due to overweight ****versus* satisfied***			Dissatisfaction due to underweight ****versus* satisfied***			Dissatisfaction due to overweight ****vrsus* satisfied***		
		cOR^*^	95%CI^†^	P^‡^	cOR^*^	95%CI^†^	P^‡^	aOR^§^	95%CI^†^	P^‡^	aOR^§^	95%CI^†^	P^‡^
**Sex**									.				
	Girls	0.89	[0.77; 1.74]	0.138	1.23	[1.07; 1.40]	0.002	0.88	[0.75; 1.02]	0.095	**1.24**	[1.09; 1.41]	0.001
	Boys	ref			ref								
**Age**													
		1.06	[0.98; 1.15]	0.156	0.93	[0.86; 0.99]	0.032	----	----	----	----	----	----
**ABEP** ^||^													
		0.99	[0.98; 1.00]	0.006	1.00	[1.00; 1.01]	0.081	**0.99**	[0.98; 1.00]	0.003	**1.01**	[1.00; 1.01]	0.043
**City**													
	Eusébio	1.06	[0.89; 1.27]	0.492	1.12	[0.99; 1.31]	0.188	----	----	----	----	----	----
	Fortaleza	1.16	[0.99; 1.36]	0.071	0.97	[0.84; 110]	0.593	----	----	----	----	----	----
	São Paulo	ref			ref								
**Drug use**													
	Alcohol	1.06	[0.92; 0.12]	0.390	1.07	[0.95; 1.21]	0.262	----	----	----	----	----	----
	Binge drinking	1.15	[0.97; 1.37]	0.105	1.02	[0.87; 1.19]	0.809	----	----	----	----	----	----
	Smoking	1.27	[0.98; 1.65]	0.067	0.91	[0.72; 1.14]	0.406	----	----	----	----	----	----
	Inhalants	1.24	[1.00; 1.53]	0.047	1.07	[0.87; 1.32]	0.507	----	----	----	----	----	----
	Marijuana	1.36	[1.03; 1.78]	0.029	0.88	[0.68; 1.14]	0.338	**1.39**	[1.05; 1.81]	0.020	0.87	[0.67; 1.13]	0.236
	Cocaine	1.11	[0.34; 3.61]	0.868	0.93	[0.31; 2.11]	0.901	----	----	----	----	----	----
	Weight-loss drugs	1.15	[0.58; 2.31]	0.687	1.42	[0.76; 2.65]	0.267	----	----	----	----	----	----

*cOR = Crude Odds Ratio; ^†^CI = Confidence interval; ^‡^p value; ^§^Adjusted Odds Ratio; ^||^ABEP = Brazilian Association of Market Research Companies

## Discussion

The most relevant finding of the study is that data refer to a representative sample of adolescents, an initial phase of this stage of life, from a middle-income country, showing a high prevalence of body dissatisfaction. In general, gender and socioeconomic class are two factors highly associated with body dissatisfaction, but with opposite effects, depending on the type of dissatisfaction.

The results highlight prevalence of dissatisfaction due to overweight, showing a trend also found in other studies about body dissatisfaction, ranging from 44% to 83% (dissatisfaction due to overweight) and from 1.7% to 37% (dissatisfaction due to underweight)^21^ ^,^ ^28^ ^-^ ^29^. Some factors can influence this problem among adolescents, such as sex, anthropometric measurements, unsatisfactory meal pattern, relationship with peers (third party provocation), and social media^30^.

In Brazil, however, this topic deserves attention among adolescents, since the impact of body dissatisfaction can lead to a reduction in quality of life and affect biopsychosocial aspects, such as weaknesses of mental health, eating disorders, use of anabolic steroids, and even higher chances of suicidal thoughts and ideation^31^ ^-^ ^32^. For the actions of school nurses, this information is valuable to promote adolescent care management.

Most adolescents were dissatisfied with their body due to overweight, especially girls. In agreement with these findings, Chinese adolescents also prefer smaller bodies^33^, and other studies in Brazil have identified that while boys overestimate their body image, girls want smaller silhouettes^28^ ^,^ ^34^ ^-^ ^35^. Clear influence of social media on the body image was observed, generating, in most cases, dissatisfaction due to a desire to have an ideal physical standard (female thin body and male muscular body), causing fear of possible rejection and psychological distress^36^ ^-^ ^39^. With the advent of social media such as Instagram, TikTok, and Facebook, the search for perfect bodies and lifestyles has increased considerably through user profiles called *fitspiration*, which has put the emotional health of teenagers at risk. Studies indicate that certain profiles on these social media can lead to negative mood and decreased body satisfaction, especially among girls^40^.

Identity construction is noticeable in adolescence, and in this period adolescents may suffer social pressure for behaviors that are dangerous to health. Then, it is important to learn about the context of vulnerability inherent to this phase, emphasizing that body dissatisfaction is more common among girls, as they are more subject to the alcohol use and smoking when they have negative images of themselves^41^.

In addition to the differences between genders, our findings point to influences of the socioeconomic status, as the chances of dissatisfaction due to underweight are lower among higher social classes, while dissatisfaction due to overweight is higher. One explanation is that lower socioeconomic classes show higher proportion of adolescents who want larger silhouettes, a fact that highlight the vulnerability of this population, particularly regarding food insecurity of families^42^ ^-^ ^43^.

Another important aspect is that eating patterns and physical activity practice are associated with body dissatisfaction as adolescents usually adopt unhealthy diets, and/or do not practice physical activity properly^44^ or skip meals^45^. In Brazil, regardless of the region, the eating habits of adolescents mostly involve fatty foods, fast foods, foods with sugar, disregarding the importance of nutrients^46^.

Regarding body dissatisfaction due to underweight, this study showed that adolescents who used marijuana were more likely to present this condition. Then, the literature^47^ showed that body dissatisfaction at 14 years of age in girls and boys was a predictor of marijuana use at 21. As this study analyzed cross-sectional data, marijuana cannot be considered a predictor of dissatisfaction or whether dissatisfaction led to marijuana use, but it deserves a deeper evaluation about its use and practices adopted by young people. From a pharmacological point of view, this finding could be explained by the fact that adolescents who are dissatisfied due to underweight may think that marijuana would increase their appetite and, consequently, help them gain weight. This perception would be based on the potential effect of marijuana in cases of anorexia, leading to an increase in weight^48^.

Although the results do not indicate a significant association of alcohol and other drugs (smoking, inhalants, cocaine, and weight-loss drugs) with body dissatisfaction, the fact that 35.67% of the participants had already tried alcohol and 19.28% had episodes of binge drinking, which refers to heavy drinking in a short period (alcoholic consumption of five or more drinks over two hours), shows the importance of studies assessing the problem of alcohol use in adolescence, with care and health education initiatives that actually bring changes in the lives of adolescents. Recent studies conducted in countries such as the United States and Brazil highlight that binge drinking among male and female adolescents can have serious consequences for society and the health system, with a significant increase in hospital admission rates, involvement in traffic accidents, family violence, and other negative outcomes^48^ ^-^ ^49^.

In addition, body dissatisfaction is directly related to the consumption of alcohol in life (current and excessive consumption), as the association is common, especially regarding excessive consumption, which may be a coping mechanism or a weight change strategy^5^.

In relation to smoking and use of inhalants, cocaine and weight-loss drugs, the use of inhalants was more prevalent. Inhalants are central nervous system depressant substances that can be breathed in (through the mouth or nose), with volatile and flammable characteristics, commonly associated with solvents (examples: glue, nail polish, *lança-perfume*, acetone, *loló*)^6^. In adolescence, inhalants impair growth, affect metabolism and food intake, change food preferences and glucose metabolism and skeletal muscle adiposity, in addition to predisposing users to withdrawal syndrome, which is directly related to body image^50^.

Then, the results of our study provide evidence to adolescent health professionals to consider body dissatisfaction as a public health problem, and it is important to promote early interventions and prevent risky behaviors. In Brazil, the culture related to alcohol consumption is so permissive in adolescence that, when combined with poor control in the sale of alcoholic drinks, requires investments in studies to assess these vulnerabilities.

The School Health Program (PSE) has an important role in Brazil; however, weaknesses are observed in the implementation of policies and programs to reduce the high rates of alcohol and drug use at early ages, reinforcing the need for specific strategies for this stage of life. It should be noted that investments in health education programs that promote the health of adolescents in the school environment help reduce smoking, episodes of heavy drinking, and drug use, such as marijuana, as observed in European countries^18^.

Study limitations include the fact that it covered only three Brazilian cities, so it should be analyzed with caution regarding the representation of the Brazilian population and its great diversity in the geographic area. In addition, the questionnaires were applied in a school environment, which can be influenced by the environment and peers, and even if teachers were not present in the classroom during data collection, it could suggest some type of recrimination or judgment in the imagination of adolescents.

## Conclusion

Our study showed a high prevalence of body dissatisfaction in adolescents who have just started this phase, with an emphasis on dissatisfaction due to overweight, presented in different ways when comparing boys and girls, and different social classes. Then, this study may contribute to reflections of health and education professionals on the implementation of educational health care actions addressing body image, relating it to various subjective and behavioral attributes that can affect the health of adolescents, whether in the community or school environment.

In addition, public prevention programs and policies that encourage positive perceptions of self-image and better self-acceptance among adolescents must have special attention, considering the specificities of each individual, in order to promote healthy youth and minimize negative outcomes, such as abuse of alcohol and other drugs.
